# Study on the factors influencing lens opacity among medical radiation workers exposed to long-term low-dose ionizing radiation

**DOI:** 10.3389/fmed.2025.1600355

**Published:** 2025-05-14

**Authors:** Anfang Ye, Jianing Li, Xiaoji Hao, Zhongjun Lai, Jiadi Guo, Yiyao Cao, Shunfei Yu, Zhiqiang Xuan

**Affiliations:** ^1^Department of Occupational Medicine, The First Affiliated Hospital, Zhejiang University School of Medicine, Hangzhou, Zhejiang, China; ^2^School of Public Health, Suzhou Medical College of Soochow University, Suzhou, Jiangsu, China; ^3^Department of Occupational Health and Radiation Protection, Zhejiang Provincial Center for Disease Control and Prevention, Hangzhou, Zhejiang, China

**Keywords:** low-dose, ionizing radiation, radiological workers, lens, opacity

## Abstract

**Background:**

Lens damage induced by occupational exposure to ionizing radiation has been extensively studied by radiation workers. This study aimed to investigate the factors influencing lens opacity in radiologists exposed to low-dose ionizing radiation.

**Methods:**

Medical examination data of 1,456 radiological workers who underwent occupational health checkups between January 2023 and December 2024 were collected, along with their total personal radiation dose over a 10-year period from 2015 to 2024. The relationship between lens opacity and influencing factors such as sex, age, radiation dose, occupational type, and duration of radiation work was analyzed using multivariate logistic regression.

**Results:**

Among the 1,456 radiological workers, 105 cases of lens opacity were detected, with a prevalence rate of 7.21%. The majority of lens opacities were located in the posterior subcapsular region, accounting for 52 cases (49.52%, 52/105). The prevalence of lens opacity revealed a linear increasing trend with age and years of service. In addition, the proportion of lens opacity gradually increased with increasing total and annual radiation doses. Age, occupational type, and total radiation dose were associated with posterior subcapsular opacity. Age and total radiation dose were regarded as independent risk factors [age Odds Ratio (OR), 1.068; 95% confidence interval (CI), 1.035–1.103; total dose OR, 1.111; 95% CI, 1.033–1.194]. The three occupational types with the highest prevalence were nuclear medicine (6/51), radiation therapy (14/240), and interventional radiology (18/340).

**Conclusion:**

The prevalence of lens opacity among radiological workers was associated with age, radiation dose, occupational type, and duration of radiation work. Nuclear medicine poses the highest risk for posterior subcapsular opacity.

## 1 Introduction

Lens opacity, also known as cataract, is characterized by the clouding of the eye’s lens and is a leading cause of blindness (1, 2). In the United States, 22% of adults aged 40 and older have cataracts, with a certain genetic risk associated with the condition (3, 4). Studies have shown that individuals with diabetes have a higher risk of developing lens opacity than those without diabetes (5). In addition, evidence suggests that lens opacity is associated with genetic disorders, including Down syndrome and Wilson disease (6). Recently, several countries and regions have made significant progress in addressing the issue of lens opacity. However, lens opacity remains the leading cause of vision impairment worldwide (7–9).

The biologically damaging effects of long-term low-dose ionizing radiation exposure on radiological workers have attracted widespread attention from society (10–13). The lens is one of the most sensitive organs to ionizing radiation (14–16). Clinically, lens opacity in radiological workers is used to monitor and assess the health impact of occupational ionizing radiation exposure (2, 17). An increasing number of studies have examined the effects of low-dose ionizing radiation on the lens. For instance, one study has found that lens opacity and vision-impairing cataracts can result from relatively low-dose radiation exposure (1 Gy or below) (15). Another study among physicians indicated that long-term exposure to occupational radiation could lead to cortical and posterior subcapsular lens opacities (18). Despite adequate evidence of the effect of low-dose ionizing radiation exposure on lens opacity, further research is needed to understand the long-term (>10 years) effects of low-dose ionizing radiation on the lenses of radiation workers.

This study aimed to examine the epidemiological characteristics of lens opacity in medical radiological workers exposed to low-dose ionizing radiation by analyzing their occupational health examination data and historical personal dose monitoring records.

## 2 Materials and methods

### 2.1 Participants and study design

This study collected data from 1,456 radiological workers who underwent occupational health examinations at provincial and municipal medical institutions between January 2023 and December 2024. Among them, 870 were male and 586 were female, with ages ranging from 24 to 83 years. The median age was 39 years (interquartile range: 33, 47). The duration of radiation work ranged from 1 to 49 years, with a median of 8 years (interquartile range 4, 15). The distribution of occupational types included 801 cases in diagnostic radiology, 23 cases in dental radiology, 51 cases in nuclear medicine, 240 cases in radiation therapy, and 340 cases in interventional radiology.

### 2.2 Personal dose monitoring for external exposure

Based on data from the National Radiological Health Information Platform, the cumulative total personal radiation dose (total dose) of radiological workers over a 10-year period, from 2015 to 2024, was collected and explored. The annual average dose (annual dose) was calculated by dividing the total dose by the number of years of service.

### 2.3 Ophthalmic examination and assessment

In this study, the following examinations were conducted: (1) corrected vision test; (2) intraocular pressure measurement; and (3) slit-lamp microscopy of the cornea, anterior chamber, iris, and fundus. After the preliminary exclusion of glaucoma, the pupils were dilated three times using compound tropicamide eye drops, with an interval of 5 min between each application. Lens conditions in both eyes were then examined using a slit-lamp microscope. In patients with lens abnormalities, lens opacity was observed and classified according to the Lens Opacities Classification System III. Lens opacities were categorized and graded as cortical, nuclear, and posterior subcapsular.

### 2.4 Health examination personnel questionnaire survey

The questionnaire was designed by a research team and distributed by trained investigators; with participants completing it independently. The content included the following: (1) basic Information: age, years of service, occupational type, presence of diabetes, medication use, smoking, alcohol consumption, ultraviolet radiation exposure, eye surgery, chemical eye injuries, contact lens use, history of radiotherapy, and long-term medication use. (2) Ocular Symptoms: presence of dry eyes, burning sensation, foreign body sensation, photophobia, tearing, blurred vision, or eye fatigue. (3) Protective Measures: Assessment of whether the working environment of radiological workers has adequate protective measures and whether they wear protective eyewear or other shielding measures. Individuals with lens opacities caused by other reasons were excluded from the study.

### 2.5 Statistical analysis

Statistical analysis was performed using SPSS software version 25.0; Chicago, IL, United States. Continuous variables with a normal distribution are expressed as means and standard deviations, whereas those with a skewed distribution are depicted as medians with interquartile ranges. Categorical variables are presented as frequencies and percentages. Comparisons between the groups were conducted using the *χ*^2^ test, Fisher’s exact test, or trend test. Univariate and multivariate logistic regression analyses were used to identify factors influencing lens opacity. A *p*-value < 0.05 was considered statistically significant.

## 3 Results

### 3.1 Basic information

Over the 10-year period from 2015 to 2024, the average total radiation dose per radiological worker was 1.10 mSv (0.47, 2.30), while the average annual dose per worker was 0.18 mSv (0.10, 0.32). The highest total individual dose was 27.02 mSv, and the lowest was 0.01 mSv.

### 3.2 Lens opacity examination results

**Figure 1:**
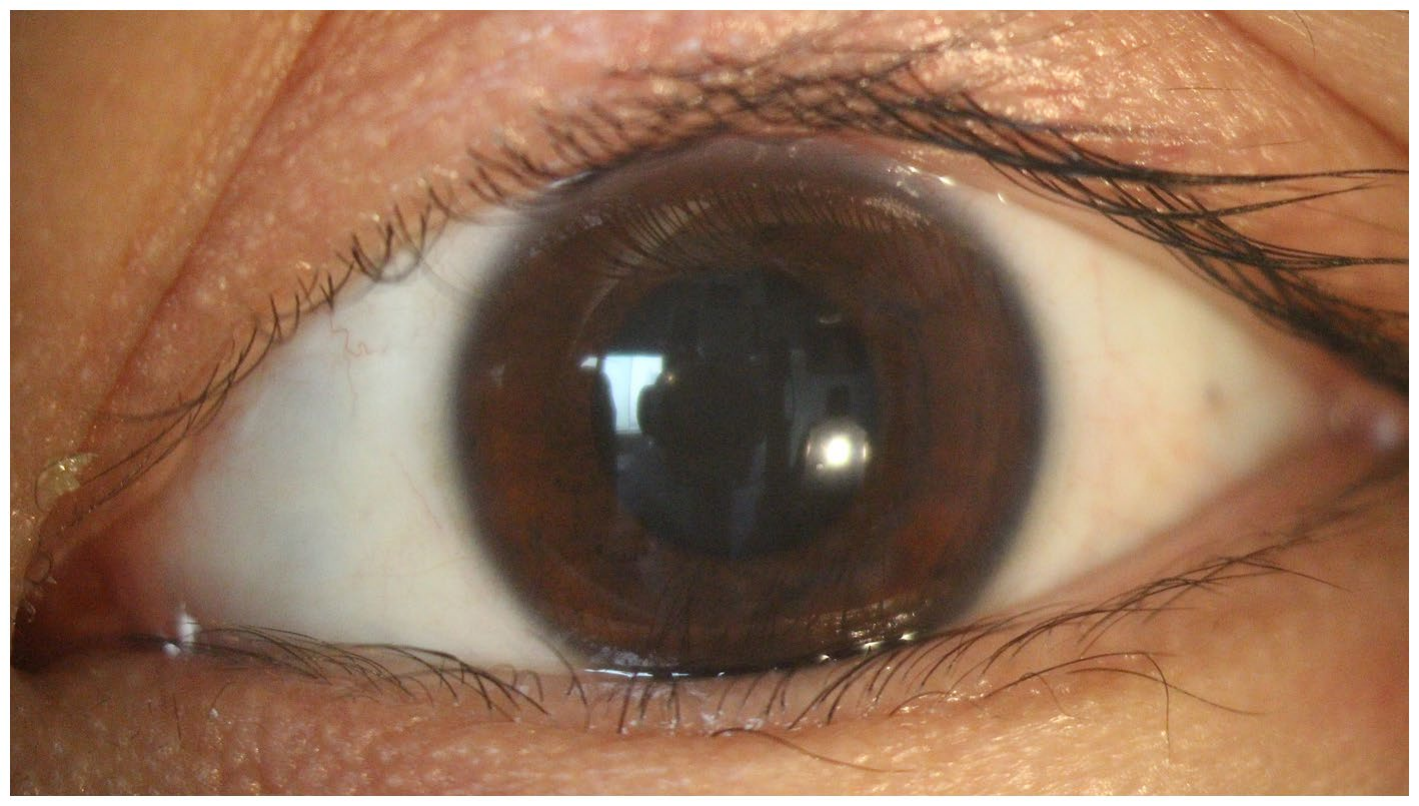
Normal lens.

**Figure 2:**
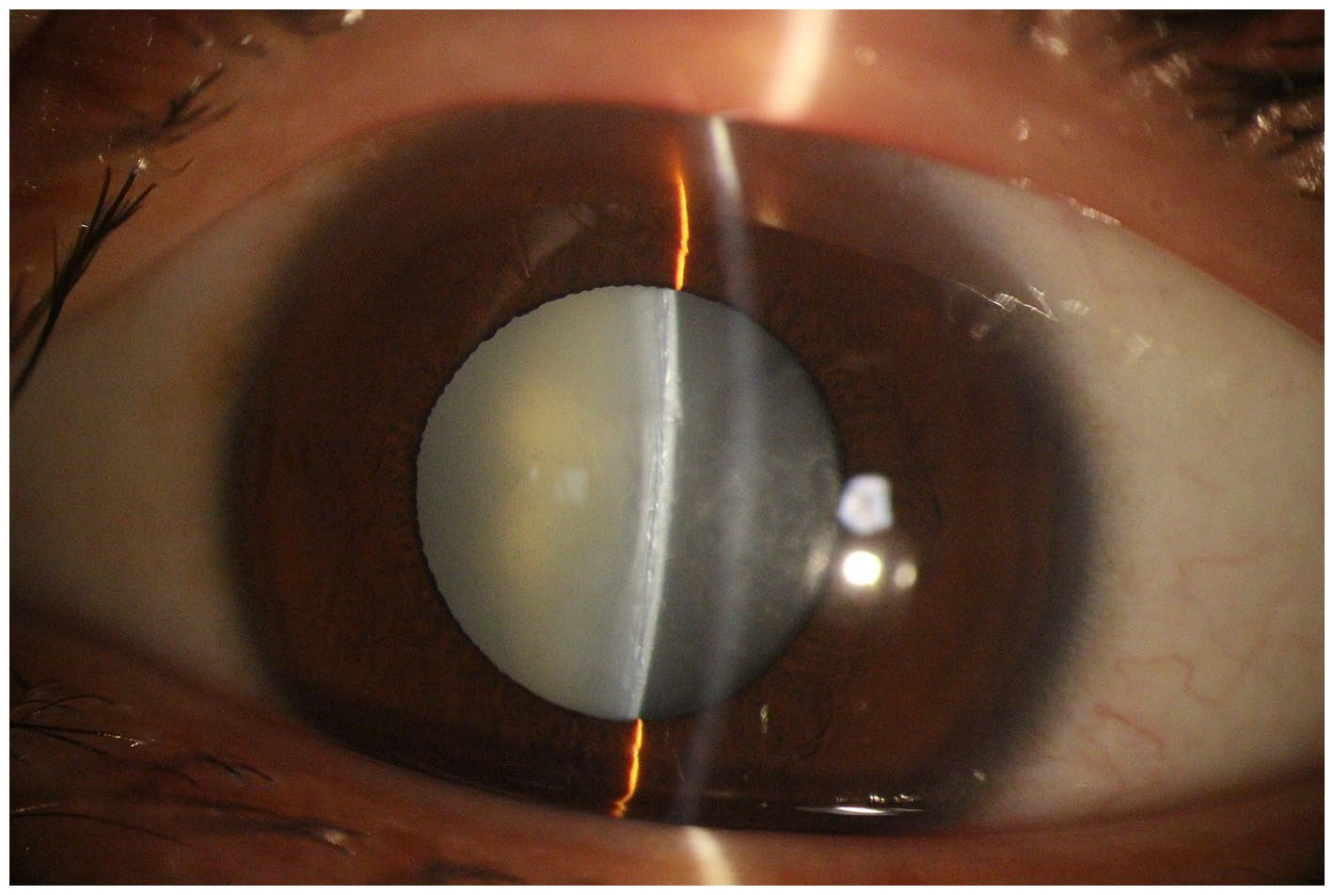
Cortical cataract.

**Figure 3:**
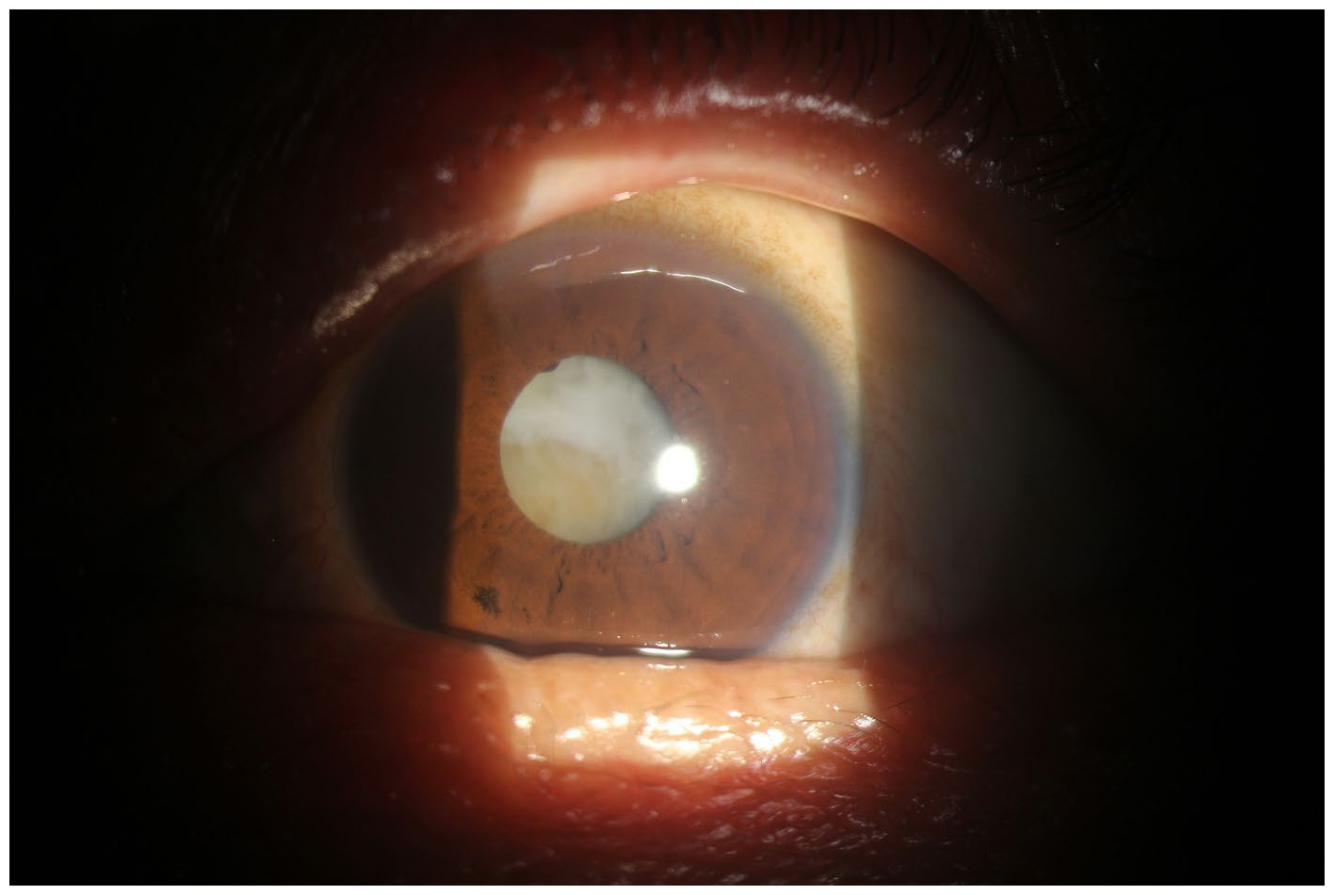
Nuclear cataract.

**Figure 4:**
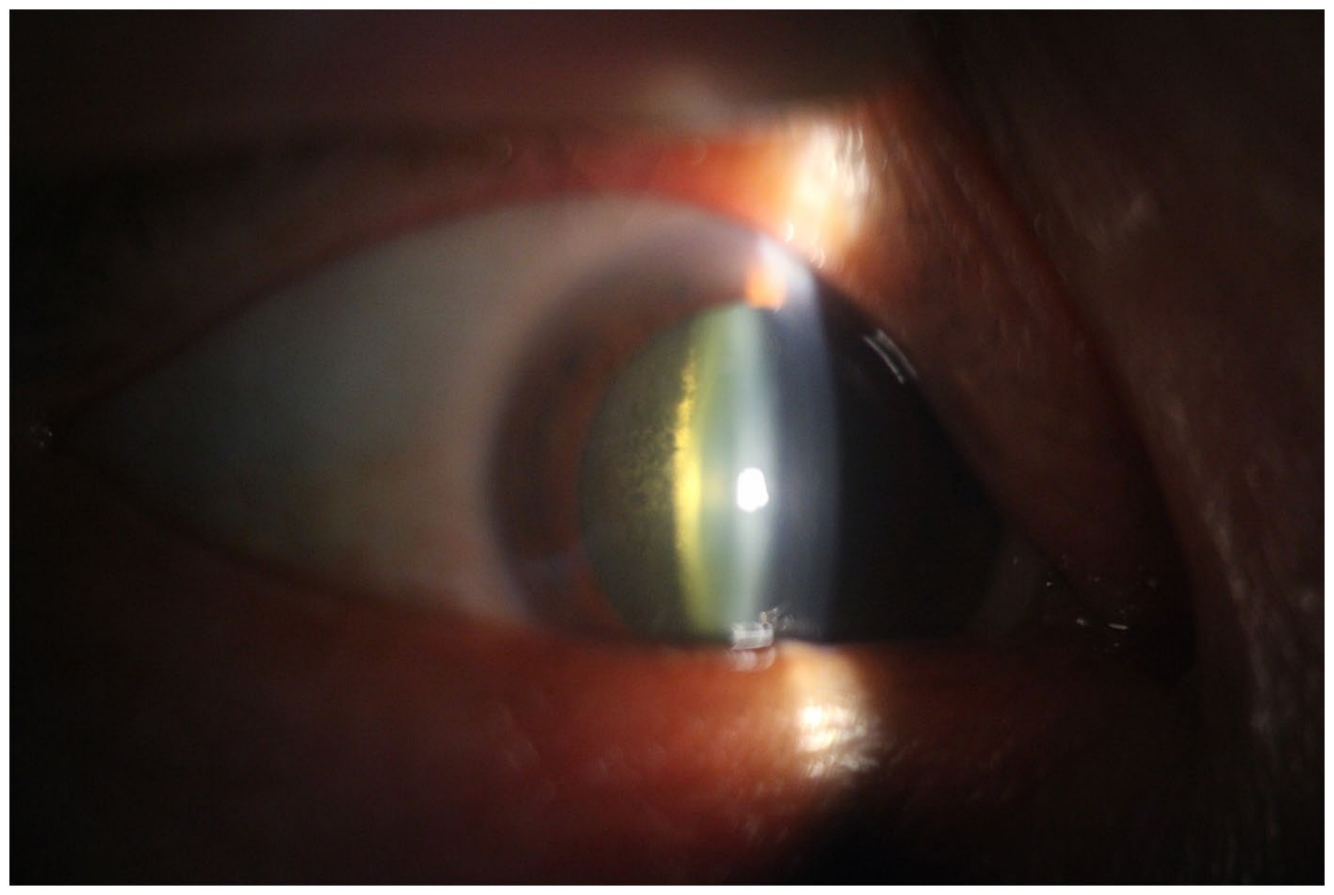
Posterior subcapsular cataract.

A total of 105 cases of lens opacity were detected in this study, with a prevalence of 7.21%. Among these cases, 52 (49.52%) had posterior subcapsular opacity, 42 (40.00%) had cortical opacity, and 11 (10.48%) had nuclear opacity (Figures 1–4).

#### 3.2.1 Analysis of factors influencing lens opacity

No statistically significant differences were observed in the impact of sex or occupational type on lens opacity (*p* > 0.05). However, the prevalence of lens opacity revealed a linear increasing trend with age and years of service (trend *χ*^2^: age = 158.56, years of service = 119.06, *p* < 0.001). Additionally, significant differences were observed in lens opacity rates among workers with different total radiation doses and annual radiation doses (*χ*^2^ total radiation dose = 46.90, annual radiation dose = 15.82, *p* < 0.001). The proportion of patients with lens opacity gradually increased with increasing total and annual radiation doses (Table 1).

**Table 1:** Basic information and opacity of the lens in 1,456 radiation workers.

Variable	Number	Cases	Rate (%)	*χ*^2^ value	*p*-value	Trend *χ*^2^ value	*p*-value
Sex				0. 79	0. 376	0. 78	0. 376
Male	870	67	7.71				
Female	586	38	6.48				
Age				158.56	<0.001	111.97	<0.001
<30	159	2	1.26				
30 ~ <40	574	17	2.96				
40 ~ <50	421	20	4.75				
50 ~ <60	251	45	17.93				
60~	51	21	41.18				
Working age (year)				119.06	<0.001	78.69	<0.001
<5	401	11	2.74				
5 ~ <10	384	18	4.69				
10 ~ <20	432	26	6.02				
20 ~ <30	142	18	12.68				
30~	97	32	32.99				
Type of work				2.34	0.674		
Radiodiagnostics	801	56	6.99				
Dental radiology	24	1	4.17				
Nuclear medicine	51	6	11.76				
Radiotherapeutics	240	15	6.25				
Interventional radiology	340	27	7.94				
Total dose (mSv)				46.90	<0.001	44.42	<0.001
<1	673	23	3.42				
1 ~ <5	674	61	9.05				
5 ~ <10	86	14	16.28				
10~	23	7	30.43				
Annual dose (mSv/a)				15.82	<0.001	21.07	<0.001
<0.5	1,296	83	6.40				
0.5 ~ <1	126	13	10.32				
1 ~ <2	27	6	22.22				
2~	7	3	42.86				

#### 3.2.2 Detection results of different types of lens opacity by occupational type

No statistically significant difference was observed in the prevalence of nuclear opacity among occupational types (*p* > 0.05). Nevertheless, statistically significant differences were observed in the prevalence of cortical and posterior subcapsular opacities among occupational types (*p* < 0.05), as shown in Table 2.

**Table 2:** Number and proportion of ocular lens opacity detected in 1,456 radiation workers of different occupational categories (%).

Type of work	Number	Mesocortex opacity		Nucleogenicity opacity		Subcapsule opacity	
		Cases	Rate (%)	Cases	Rate (%)	Cases	Rate (%)
Radiodiagnostics	801	35	4.37	7	0.87	14	1.75
Dental radiology	24	1	4.17	0	0.00	0	0.00
Nuclear medicine	51	0	0.00	0	0.00	6	11.76
Radiotherapeutics	240	1	0.42	0	0.00	14	5.83
Interventional radiology	340	5	1.47	4	1.18	18	5.29
Total	1,456	42	2.89	11	0.76	52	3.57
Fisher’s *P*-value		0.002		0.537		<0.001	

### 3.3 Univariate logistic regression analysis results of lens opacity

Using various types of lens opacity (including cortical, posterior subcapsular, and nuclear opacity) as dependent variables, univariate logistic regression analysis was conducted. Independent variables included sex, age, years of service, occupational type, total radiation dose, and annual radiation dose. Age, years of service, total radiation dose, and annual radiation dose were associated with lens, cortical, and posterior subcapsular opacities (all *p* < 0.05). The occupational type was related to posterior subcapsular and cortical opacity (both *p* < 0.05) but not to nuclear opacity (*p* > 0.05) (Table 3).

**Table 3:** Results of univariate logistic regression analysis of opacification of the eye lens in 1,456 radiation workers.

Turbidity type	Factor	*B*	SE	Wald	Sig	Exp(B)	95%CI for Exp(B)	
							Lower	Upper
Lens opacity	Sex	0.186	0.211	0.783	0.376	1.205	0.797	1.820
	Age	0.118	0.012	105.580	<0.001	1.126	1.101	1.151
	Working age	0.088	0.009	88.828	<0.001	1.092	1.072	1.112
	Type of work							
	Radiodiagnostics			2.466	0.651			
	Dental radiology	−0.503	1.032	0.238	0.626	0.605	0.080	4.569
	Nuclear medicine	0.573	0.456	1.579	0.209	1.774	0.725	4.337
	Radiotherapeutics	−0.120	0.301	0.159	0.690	0.887	0.492	1.598
	Interventional radiology	0.138	0.244	0.319	0.572	1.148	0.712	1.851
	Total radiation dose	0.155	0.028	30.405	<0.001	1.168	1.105	1.234
	Annual dose	1.131	0.264	18.283	<0.001	3.097	1.845	5.201
Mesocortex	Sex	0.422	0.338	1.552	0.213	1.524	0.785	2.958
	Age	0.161	0.019	69.915	<0.001	1.175	1.131	1.220
	Working age	0.112	0.014	63.629	<0.001	1.119	1.088	1.150
	Type of work							
	Radiodiagnostics			9.797	0.044			
	Dental radiology	−0.033	1.037	0.001	0.975	0.968	0.127	7.385
	Nuclear medicine	−18.145	5991.614	0.000	0.998	0.000	0.000	–
	Radiotherapeutics	−2.358	1.017	5.376	0.020	0.095	0.013	0.694
	Interventional radiology	−1.079	0.483	4.992	0.025	0.340	0.132	0.876
	Total radiation dose	0.139	0.039	13.067	<0.001	1.150	1. 066	1.240
	Annual dose	0.919	0.404	5.187	0.023	2.508	1.137	5.532
Nucleogenicity	Sex	1.922	1.050	3.348	0.067	6.833	0.872	53.530
	Age	0.238	0.046	26.617	<0.001	1.268	1.159	1.388
	Working age	0.185	0.036	27.173	<0.001	1.203	1.122	1.290
	Type of work							
	Radiodiagnostics			0.238	0.993			
	Dental radiology	−16.535	8569.170	0.000	0.998	0.000	0.000	–
	Nuclear medicine	−16.535	5991.614	0.000	0.998	0.000	0.000	–
	Radiotherapeutics	−16.535	2679.531	0.000	0.995	0.000	0.000	–
	Interventional radiology	0.308	0.630	0.238	0.626	1.360	0.395	4.679
	Total radiation dose	0.172	0.058	8.822	0.003	1.187	1.060	1.330
	Annual dose	1.418	0.582	5.940	0.015	4.127	1.320	12.904
Subcapsule	Sex	−0.227	0.284	0.639	0.424	0.797	0.457	1.390
	Age	0.074	0.015	24.159	<0.001	1.077	1.045	1.109
	Working age	0.051	0.014	13.772	<0.001	1.052	1.024	1.081
	Type of work							
	Radiodiagnostics			19.035	0.001			
	Dental radiology	−17.229	8569.170	0.000	0.998	0.000	0.000	–
	Nuclear medicine	1.959	0.512	14.673	<0.001	7.095	2.603	19.337
	Radiotherapeutics	1.197	0.386	9.644	0.002	3.311	1.555	7.049
	Interventional radiology	1.118	0.363	9.512	0.002	3.060	1.503	6.229
	Total radiation dose	0.144	0.035	17.189	<0.001	1.155	1.079	1.236
	Annual dose	1.149	0.338	11.564	0.001	3.156	1.627	6.122

Analysis of lens opacity had a sample size of 1,456. The analysis of cortical opacity had a sample size of 1,392 (1,350 cases without lens opacity and 42 cases with cortical opacity). The analysis of nuclear opacity included a sample size of 1,361 (1,350 cases without lens opacity and 11 cases with nuclear opacity). The analysis of posterior subcapsular opacity had a sample size of 1,402 (1,350 cases without lens opacity and 52 cases with posterior subcapsular opacity).

### 3.4 Multivariate logistic regression analysis results of lens opacity

Multivariate unconditional regression analysis was performed using lens opacity, cortical opacity, nuclear opacity, and posterior subcapsular opacity as dependent variables. Independent variables included age, years of service, occupational type, total radiation dose, and annual radiation dose—factors identified as associated with various types of lens opacity in the univariate logistic regression analysis.

The results indicated that age and total radiation dose were factors influencing lens opacity (*p* < 0.05). Age (OR, 1.122; 95% CI, 1.096–1.148) and total radiation dose (OR, 1.107; 95% CI, 1.041–1.177) were identified as risk factors, with the risk of lens opacity increasing as age and total radiation dose increases.

Factors influencing cortical lens opacity included age and occupational type (*p* < 0.05). Age (OR, 1.174; 95% CI, 1.130–1.219) was a risk factor, indicating that the risk of cortical opacity increased with age. Using diagnostic radiology as the reference category, radiation therapy (OR, 0.099; 95% CI, 0.013–0.744) and interventional radiology (OR, 0.242; 95% CI, 0.088–0.662) revealed significantly lower risks of cortical lens opacity compared to diagnostic radiology (*p* < 0.05). Specifically, the risk of cortical opacity in radiation therapy workers was 0.099 times that of diagnostic radiology workers, and the risk in interventional radiology workers was 0.242 times that of diagnostic radiology workers. Thus, compared to radiation therapy and interventional radiology, diagnostic radiology posed a higher risk factor for cortical opacity.

Research analysis indicated that age and annual dose were independent risk factors influencing the nuclear opacity of the lens (*p* < 0.05). Both age (OR: 1.210, 95% CI: 1.079–1.361) and annual dose (OR: 6.774, 95% CI: 1.385–33.144) were risk factors. This suggests that the risk of nuclear opacity increases with age and annual dose.

Factors influencing posterior subcapsular opacity of the lens included age, type of work, and total dose (*p* < 0.05). Both age (OR: 1.068, 95% CI: 1.035–1.103) and total dose (OR: 1.111, 95% CI: 1.033–1.194) are risk factors. This indicates that the risk of posterior subcapsular opacity increases with age and total dose. Using diagnostic radiology as the reference category, occupational type of work also revealed significant differences: nuclear medicine (OR: 5.638, 95% CI: 1.967–16.157), radiotherapy (OR: 3.444, 95% CI: 1.580–7.507), and interventional radiology (OR: 2.716, 95% CI: 1.297–5.687) were all associated with a significantly higher risk of posterior subcapsular opacity compared with radiology (*p* < 0.05). Specifically, the risk of posterior subcapsular opacity in nuclear medicine workers was 5.638 times that in radiology workers, the risk in radiotherapy workers was 3.444 times that in radiology workers, and the risk in interventional radiology workers was 2.716 times that in radiology workers, as shown in Table 4 headings.

**Table 4:** Logistic regression analysis of multiple factors influencing lens opacity.

Turbidity type	Factor	*B*	SE	Wald	Sig	Exp(B)	95%CI for Exp(B)	
							Lower	Upper
Lens opacity	Age	0.115	0.012	94.306	<0.001	1.122	1.096	1.148
	Working age	0.186	0.052	1.477	0.226	0.781	0.425	1.291
	Total radiation dose	0.102	0.031	10.544	0.001	1.107	1.041	1.177
	Annual dose	0.234	0.058	1.431	0.211	0.769	0.439	1.352
Mesocortex	Age	0.160	0.019	68.311	<0.001	1.174	1.130	1.219
	Working age	0.386	0.062	1.342	0.206	0.752	0.465	1.391
	Type of work							
	Radiodiagnostics			12.601	0.013			
	Dental radiology	0.767	1.185	0.418	0.518	2.152	0.211	21.961
	Nuclear medicine	−17.656	5645.931	0.000	0.998	0.000	0.000	–
	Radiotherapeutics	−2.311	1.028	5.049	0.025	0.099	0.013	0.744
	Interventional radiology	−1.420	0.514	7.621	0.006	0.242	0.088	0.662
	Total radiation dose	0.416	0.056	1.384	0.176	0.862	0.612	1.287
	Annual dose	0.397	0.106	1.021	0.297	0.648	0.305	1.228
Nucleogenicity	Age	0.191	0.060	10.132	0.001	1.210	1.076	1.361
	Working age	0.067	0.042	2.477	0.116	1. 069	0.984	1.161
	Total radiation dose	0.153	0.087	2.045	0.128	0.897	0.721	1.134
	Annual dose	1.913	0.812	5.577	0.018	6.774	1.385	33.144
Subcapsule	Age	0.066	0.016	16.473	<0.001	1.068	1.035	1.103
	Working age	0.346	0.101	1.135	0.258	0.607	0.545	1.231
	Type of work							
	Radiodiagnostics			15.100	0.004			
	Dental radiology	−16.736	8282.783	0.000	0.998	0.000	0.000	–
	Nuclear medicine	1.729	0.537	10.366	0.001	5.638	1.967	16.157
	Radiotherapeutics	1.237	0.398	9.680	0.002	3.444	1.580	7.507
	Interventional radiology	0.999	0.377	7.019	0.008	2.716	1.297	5.687
	Total radiation dose	0.105	0.037	8.049	0.005	1.111	1.033	1.194
	Annual dose	0.286	0.115	1.253	0.221	0.797	0.623	1.358

## 4 Discussion

In the present study, the highest total individual dose, highest average annual dose, and annual dose per capita among 1,456 medical radiation workers over a 10-year period were well below the dose thresholds for tissue response. These findings align with the characteristics of long-term, low-dose ionizing radiation exposure (19, 20). The lens turbidity rates among medical radiation workers examined between 2023 and 2024 were similar to the findings of previous studies by this research group and those from other provinces. Differences in lens turbidity rates according to age group, years of service, total dose, and average annual dose were statistically significant. Notably, the lens turbidity rate increased linearly with age and years of service. In addition, as the total radiation dose and annual average dose increased, the proportion of lens opacity also gradually rose (21–23).

Although this study found no statistically significant differences in the overall prevalence of lens opacity among different types of work when the specific types of lens opacity were further subcategorized, particularly posterior subcapsular opacity, which is closely associated with radiation exposure. The differences between the different types of work were statistically significant. The top three studies with the highest detection rates of posterior subcapsular opacity were nuclear medicine, radiotherapy, and interventional radiology. Through further univariate logistic regression analysis of lens opacity, factors such as age, length of service, total radiation dose, and annual average dose were found to be correlated with lens, cortical, and posterior subcapsular opacities. Low-dose ionizing radiation exposure, advanced age, long length of service, high total dose, and high annual average dose were risk factors for various types of lens opacities. While occupational type of work was associated with posterior subcapsular and cortical opacities, occupational type of work was not a relevant factor for nuclear lens opacity. Posterior subcapsular opacity is often considered a typical feature of radiation-induced cataracts. However, many studies have found that ionizing radiation is also associated with cortical opacity, whereas most studies have not identified an association with nuclear opacity. The present study supports the above findings. Multifactorial logistic regression analysis of the factors influencing lens opacity, using posterior subcapsular opacity, which is most closely related to ionizing radiation exposure, as an example, the types of work in nuclear medicine, radiotherapy, and interventional radiology were identified as risk factors and were more likely to lead to posterior subcapsular opacity. This aligns with recent research conclusions that “nuclear medicine workers have a higher specific risk of posterior subcapsular opacity following ionizing radiation exposure to the lens” (24, 25). Furthermore, numerous studies suggest that the annual effective dose for nuclear medicine radiation workers ranks the highest among all radiation-related occupations (21, 26). This may be attributed to the improper handling of radioactive drugs, inadequate management of patient waiting areas after injection, inherent radiation from patients, and a tendency for surface radioactive contamination in the workplace.

This study can provide targeted health protection measures for radiation workers, thereby reducing the incidence of occupational diseases such as cataracts. By identifying high-risk populations and key influencing factors, an early warning system can be established and intervention measures can be implemented, such as enhancing protective equipment and regular eye examinations. These findings offer evidence for governments and relevant agencies to formulate public health policies, thereby improving the health management of populations occupationally exposed to radiation.

This study primarily investigated the impact of occupational ionizing radiation exposure on lens opacity. Future research should focus on the following areas: First, further studies are required to clarify the molecular mechanisms underlying low-dose ionizing radiation-induced lens opacity, particularly the synergistic effects of genomic damage, oxidative stress, disruption of intercellular communication, and inflammatory responses (27–29). Second, large-scale epidemiological studies should be conducted to validate dose thresholds, assessing the applicability of the 0.5 Gy threshold proposed by the International Commission on Radiological Protection. This would help establish quantitative relationships between long-term low-dose cumulative exposure (e.g., occupational radiation) and lens opacity risk. Finally, leveraging the radiation-sensitive characteristics of the ocular lens, future research should prioritize the design of wearable shielding devices or nanomaterials to optimize existing radiation protection standards.

This study has several strengths. Notably, we selected the total radiation dose over a 10-year period for statistical analysis, making the research results more precise. Previous studies often faced limitations in statistically analyzing long-term total personal radiation doses, typically selecting cumulative radiation doses from a single year (30–32). However, this study has certain limitations that are worth mentioning. First, due to the close-range operations typically performed by nuclear medicine and interventional radiology workers, coupled with poor radiation protection awareness among some radiation workers and the low rate of lead glass usage, personal dose monitoring values—primarily reflecting the doses recorded by dosimeters placed inside lead protective vests on the chest–may underestimate the actual lens exposure doses. This underestimation arises from the shielding effect of lead aprons, which can lead to recorded doses that are lower than the actual exposure experienced by the lens. Additionally, inadequate radiation protection awareness and the low usage rate of lead glasses among these radiation workers further contribute to this discrepancy (33).

This study comprehensively evaluated the impact of long-term exposure to low-dose ionizing radiation on lens opacity in radiation workers by cumulatively analyzing their external exposure to personal radiation doses over a 10-year period. The findings revealed statistically significant differences in lens opacity rates based on age, length of service, total dose, and annual average dose. Furthermore, subcategorizing the types of lens opacity, and type of work, particularly nuclear medicine, was found to be associated with posterior subcapsular opacity.

## Data Availability

The original contributions presented in the study are included in the article/supplementary material, further inquiries can be directed to the corresponding author.
